# Achieving Sustainable Development Goals Through Collaborative Innovation: Evidence from Four European Initiatives

**DOI:** 10.1007/s10551-022-05193-z

**Published:** 2022-07-14

**Authors:** Laura Mariani, Benedetta Trivellato, Mattia Martini, Elisabetta Marafioti

**Affiliations:** 1 Department of Management, University of Bergamo, Via Dei Caniana, 2, 24127 Bergamo, Italy; 2 Department of Sociology and Social Research, University of Milano-Bicocca, Via Bicocca degli Arcimboldi, 8, 20126 Milan, Italy; 3 Department of Business and Law, University of Milano-Bicocca, Via Bicocca degli Arcimboldi, 8, 20126 Milan, Italy

**Keywords:** Sustainable development, Non-profit organizations, Metagovernance, Network management, Multi-stakeholder partnerships

## Abstract

The role to be played by multi-stakeholder partnerships in addressing the ‘wicked problems’ of sustainable development is made explicit by the seventeenth Sustainable Development Goal. But how do these partnerships really work? Based on the analysis of four sustainability-oriented innovation initiatives implemented in Belgium, Italy, Germany, and France, this study explores the roles and mechanisms that collaborating actors may enact to facilitate the pursuit of sustainable development, with a particular focus on non-profit organizations. The results suggest that collaborative innovations for sustainability contribute simultaneously to the fulfilment of different Sustainable Development Goals, reaching beyond their original intent, and that the value being created has the potential to reinforce such roles and mechanisms. These partnerships are prompted and managed by non-profit organizations that act as metagovernors of collaborative innovation processes as they play the roles of cultural spreaders, enablers, relational brokers, service provides, and influencers. These findings will help policy-makers and practitioners in the public and non-profit sector to identify and utilize emerging opportunities for value creation through collaborative innovation, and to better design existing and prospective collaborative efforts aimed at sustainable objectives, thereby supporting progress towards the implementation of Agenda 2030.

## Introduction

In 2015, the General Assembly of the United Nations adopted the 2030 Development Agenda. At the core of this agenda are the seventeen goals that spell out a vision for a sustainable development, including a critical role to be played by collaboration for sustainability (Schaltegger et al., 2018). These Sustainable Development Goals (SDGs) follow the previous eight Millennium Development Goals (MDGs) which, by setting up measurable and time-bound objectives, helped “promote global awareness, political accountability, improved metrics, social feedback, and public pressures.” (Sachs, 2012; p. 2206). Both the MDGs and the SDGs have their roots in a view of sustainable development that encompasses the so-called “triple bottom line approach to human wellbeing” (Sachs, 2012), integrating economic development, social inclusion, and environmental sustainability (Hammer & Pivo, 2017). The origins of this approach date back to the mid-1990s, which saw the environmental agenda broadening to include the triple bottom line, and an increasing consensus that partnerships among businesses and stakeholders are of critical importance to pursue this “much broader, and more demanding, sustainable development agenda” (Elkington, 1998). Compared to the MDGs, the SDGs similarly feature an increased emphasis on the role of partnerships between public and private sector (both for-profit and non-profit) organizations, as embodied by the 17th goal, Partnerships for the Goals. Sustainable development, in fact, is a complex concept, dealing with different temporal and spatial scales and with multiple stakeholders. As such, it requires a pluralistic approach to deal with multiple actors and multiple levels, so as to create a common vision of the planet’s future, and to resolve potential trade-offs (van Zeijil-Rozema et al., 2008). This need for an integrated approach to tackle the SDGs is, in fact, a pillar of the 2030 Agenda for Sustainable Development, where the Goals and the targets are meant to stimulate action in the following five areas: people, planet, prosperity, peace, and partnerships. A key part of the 2030 Agenda is also the Addis Ababa Action Agenda, which contains concrete measures in relation to public and private resources, international trade and aid, and a range of issues related to science, technology, innovation and capacity-building, and data, monitoring and follow-up (UN General Assembly, 2015).

Sustainable development challenges include issues such as the consequences of climate change, inequalities in access to health and education, integration of immigrants and refugees, and several others which embody the ‘wicked problems’ (Sørensen & Torfing, 2017; Torfing & Ansell, 2017) that are best addressed through forms of governance involving partnerships and multi-actors’ networks (Hofstad & Torfing, 2016; Koppenjan & Klijn, 2004; Sørensen & Torfing, 2011; Van Huijstee et al., 2007). Within the New Public Governance approach (Ansell & Torfing, 2014a, 2014b; Crosby & Bryson, 2010; Koppenjan, 2012; Osborne, 2006, 2010), such wicked problems may be fruitfully addressed through collaborative innovation, which involves constructive integration of partners’ differences and resources, and the development of new solutions that disrupt established practices (Hofstad & Torfing, 2016). Despite its promises, the collaborative innovation framework draws the attention mostly to the ‘meso’ level of the collaboration and of the arenas where collaborative processes take place, and to the ‘macro’ level of policy-making, rather than to the ‘micro’ level of the collaborating organizations. Moreover, although collaborative innovation is meant to involve both public and private (for profit and non-profit) actors, and private actors are acknowledged as its potential metagovernors, the specific role of non public actors within these processes remains relatively underexplored (Sørensen & Torfing, 2017).

In this context, we believe that a focus on the role of collaborating actors, and on non-profits in particular, is needed in order to obtain a better understanding (a) of the relevant roles, activities, and processes and how they contribute to collaborative innovation outputs; and (b) of the factors that may influence such roles, activities, and processes. Therefore, in this paper we propose that the collaborative innovation literature may be fruitfully integrated with contributions from the market/societal orientation literature (Duque-Zuluaga & Schneider, 2008; Hsieh et al., 2008; Kotler & Levy, 1969; Liao et al., 2001), stakeholder theory (Abzug & Webb, 1999; Fassin et al., 2017; Van Puyvelde et al., 2012), and grassroots sustainable innovation (Kemp et al., 1998; Seyfang & Haxeltine, 2012; Seyfang & Smith, 2007) in order to explore the roles and dynamics that allow such collaborative innovation to occur, and to translate into sustainable development improvements. In fact, an improved understanding of these dimensions at the meso and micro levels allows to better support (for instance through institutional design) and implement (e.g. thanks to greater awareness of drivers and barriers) existing and prospective collaborative endeavors aimed at sustainable objectives, and relatedly the progress towards the implementation of Agenda 2030.

In summary, multi-stakeholder collaboration is seen as a potentially crucial instrument to address the wicked problems of sustainable development both by the SDGs and by the collaborative governance and collaborative innovation literature. However, the roles and mechanisms that collaborating organizations and individuals may enact to facilitate the pursuit of sustainable development are not very clear. We therefore address this issue through our first research question: (1) How can multi-stakeholder collaboration contribute to improvements in sustainable development?

Moreover, the collaborative innovation framework explicitly concedes that not only the collaborating participants but also the metagovernor of the collaboration may be a non-profit organization (NPO) (Sørensen & Torfing, 2017). However, most studies within this literature highlight the role of a public sector organization as metagovernor: exploring the conditions where such role is played by a NPO may then be important, because it widens the scope for such critical role to be played in finding solutions to wicked problems. We focus on this issue through our second research question: (2) What role(s) do non-profit organizations play within multi-stakeholder collaborations that contribute to sustainable development?

In order to address these questions, we conducted a multiple-case study analysis of four innovation initiatives implemented across Europe. This specific empirical setting was chosen because the four initiatives result from collaborations among various actors, including NPOs in their different forms, and because they represent a befitting exemplification of how the above-mentioned ‘wicked problems’ may be addressed. We refer to these initiatives as ‘collaborative innovations for sustainability’, which group together networks of actors who generate novel bottom–up solutions that respond to the interests and values of the communities involved (Smith et al., 2014). In summary, our ultimate goal is to assess the ability and potential of these networks to address ‘wicked and unruly problems’ (Hofstad & Torfing, 2016) through collaborative innovation.

## Theoretical Background

Our theoretical framework is built on the premise that *collaboration* can play a crucial role in solving societal problems, especially within the inter-organizational partnerships that are one of the pillars of the SDGs, and which are the object of analysis in this work. As detailed below, we propose an integration of two different literature strands, so as to connect the ‘meso’ level of the collaboration / network of partners with the ‘micro’ level of the individual collaborating organizations. A meso-level perspective—grounded in New Public Governance theory (Ansell & Torfing, 2014a, 2014b; Crosby & Bryson, 2010; Koppenjan, 2012; Osborne, 2006, 2010)—sheds light on how multi-stakeholder collaboration may help to address the ‘wicked problems’ of sustainable development. A micro-level (organizational) perspective focusing on individual non-profit organizations—and grounded in the non-profit management and market orientation literature (Duque-Zuluaga & Schneider, 2008; Hsieh et al., 2008; Kotler & Levy, 1969; Liao et al., 2001)—emphasizes that an NPO needs to adopt a so-called ‘societal orientation’ to survive and fulfil its mission, with collaboration being a major component of such orientation. Joining these two perspectives highlights the positive role to be played by collaboration both for society and for individual NPOs, in the latter case through its impact on organizational performance (Duque-Zuluaga & Schneider, 2008), with the implication that increased micro-level collaborative behaviour will contribute to improved outcomes also at the meso and ultimately macro level.

### Societal Orientation and Collaboration

The micro-perspective draws on the work of those authors who have sought to adapt the concept of ‘market orientation’ from the private for-profit sector (Jaworski & Kohli, 1993; Slater & Narver, 1994) to the specificities of the private non-profit sector, while also integrating insights from stakeholder theory. This evolution emanates from the acknowledgement that a focus primarily on customers and profitability (Narver & Slater, 1990) is not appropriate for the study of NPOs, which have a wider variety of critical stakeholders in addition to customers and shareholders (Duque-Zuluaga & Schneider, 2008; Hsieh et al., 2008). Moreover, stakeholder management as a critical component of a firm’s strategy was also born in the for-profit sector (Freeman, 1984), to be later extended to a more comprehensive stakeholder theory, including an increased focus on non-profits as particular stakeholders of a focal for-profit organization (e.g. Abzug & Webb, 1999), and on NPOs being themselves the focal organizations dealing with a variety of internal and external stakeholders (e.g. Fassin et al., 2017; Van Puyvelde et al., 2012).

In this context, the ‘societal orientation’ literature (e.g. Duque-Zuluaga & Schneider, 2008; Liao et al., 2001) has emerged by pointing out what should be the key concerns for NPOs to ensure organizational performance, which include: attention to their stakeholders (e.g. users/beneficiaries, donors, employees and volunteers, competitors, and other stakeholder groups), collaborative orientation, and interfunctional coordination. Both Liao et al. (2001) and Duque-Zuluaga and Schneider (2008) consider collaborative orientation as a “crucial component of the societal orientation construct” as “(p)artnerships can ensure continuity of operation, increase the capability of solving problems, and contribute to improving the efficiency of service delivery” (Duque-Zuluaga & Schneider, 2008, p. 35). In a similar vein, Yin and Jamali (2020) suggest that value creation within social partnerships essentially depends on actors adopting a ‘partnership logic’, with the latter encompassing joint ownership of the issues being addressed, creation of synergy among opposite yet complementary goals, creative use of limited resources, and tolerance for divergent interests and unbalanced power relationships among partners.

### Multi-actor Collaboration as a Driver for Innovation

Collaborative strategies have been shown to be more advantageous than authoritative or competitive strategies especially when dealing with ‘wicked problems’, i.e. those where no definitive statement can be made about the problem itself, where stakeholders champion alternative ways to frame it and to propose solutions, and where constraints to the solving process are constantly changing (Roberts, 2000). Moreover, there is now a consensus among scholars of public and non-profit management and administration that especially fruitful are those collaborations among actors from the public, private for-profit, and private non-profit sectors, with authors referring to them with labels—just to name a few—as diverse as cross-sectoral partnerships (Huxham, 1996; Huxham & Vangen, 2005), integrated networks (Provan & Milward, 1995), networked government (Agranoff, 2007), inter-organizational partnerships for value creation (Le Pennec & Raufflet, 2018), multi-stakeholder partnerships (MacDonald et al. 2019), and social partnerships (Yin & Jamali, 2020).

Whereas the reference to complex and wicked problems often implies without making explicit the need for new solutions, Hartley (2005) explicitly highlights the existence of a relation between networked governance and *innovation*, and the roles to be played by policy makers, public managers and citizens. Eggers and Singh (2009) further reinforce this perspective when they emphasise that, in order to encourage innovation, the following strategies are especially important: *partnering* (within bilateral relationships) among government agencies, and among government, private industry, universities, and non-profits, so as to test new ideas quickly and overcome bureaucratic and financial constraints; *networking*, so as to use the innovation assets of a diverse base of organizations and individuals; and adopting *open source* innovation models that encourage several people to collaborate voluntarily to create solutions. In fact, Bommert (2010) notes that collaborative innovation is especially suited to solve persistent as well as emergent problems “because it opens the innovation cycle to a variety of actors and taps into innovation resources across borders, overcomes cultural restrictions and creates broad socio-political support for public innovation.” (Bommert, 2010, p. 29).

More recently, Torfing (2019) notes a renewed interest in the concept of collaborative innovation prompted by a growing body of literature which seeks to integrate contributions on collaborative governance (Ansell & Torfing, 2014a; Bryson et al., 2014, 2015; Cristofoli et al., 2021a, b; Emerson et al., 2012; McGuire, 2006) with those that rely more generally on theories of innovation in public sector settings (Eggers & Singh, 2009; Hartley, 2005; Sørensen & Torfing, 2011; Torfing et al., 2020; Trivellato et al., 2021). As it ascribes a crucial role to cross-sector partnerships, this literature assumes that “the participants in collaborative innovation are public and private actors that either have relevant knowledge, ideas and resources or are affected by the problem or the innovative solution and, therefore should be included in order to ensure that the problem is properly understood and the solution is feasible and solves the problem.” (Torfing, 2019, p. 4).

Collaborative innovation is, according to Hofstad and Torfing (2016), a promising means to address the ‘wicked and unruly’ problems that increasingly characterize public policy arenas, including those related to the challenges posed by sustainable development. Examples of such problems include climate change, congested cities, protection of natural resources and social inequalities in health and education (Torfing & Ansell, 2017), as well as homelessness, integration of immigrants and refugees, or gang-related crime (Sørensen & Torfing, 2017). In fact, all these examples pertain to areas of sustainable development in their social, environmental, and economic dimensions (Sachs, 2012). As such, collaborative innovation holds promise to address these issues as it “brings together a range of stakeholders from the public, for-profit, and nonprofit sectors, as well as users and citizens themselves, in interactive arenas that facilitate the cross-fertilization of ideas, mutual and transformative learning, and the development of joint ownership of new solutions.” (Hartley et al., 2013; p. 828; Trivellato et al., 2019).

### A Framework for the Study of Multi-actor Collaboration for Sustainability

**Fig. 1:**
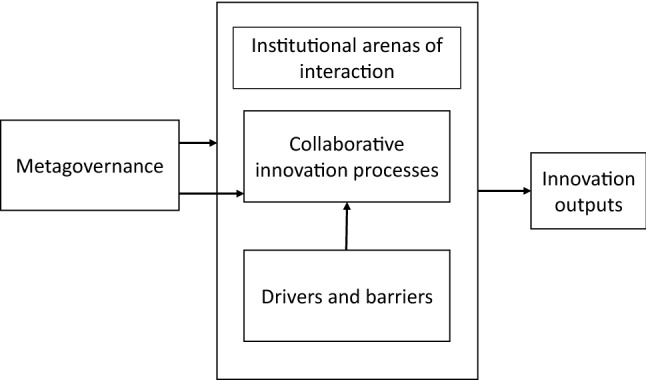
Analytical framework for the analysis of collaborative innovations for sustainability. Source: Authors’ elaboration based on Sørensen and Torfing (2011)

To assess how collaborative innovations may in practice contribute to the 2030 Agenda, we draw from a framework that was originally developed by Sørensen and Torfing (2011) for the analysis of collaborative innovation^1^ (Fig. 1), and we add contributions from the literature on grassroots innovations for sustainability (Seyfang & Haxeltine, 2012; Seyfang & Smith, 2007; Smith et al., 2014), and from the societal orientation literature (Duque-Zuluaga & Schneider, 2008; Hsieh et al., 2008; Liao et al., 2001). Our framework highlights the elements, and the interactions thereof, that lead to the generation of innovation outputs; we build on it by making explicit how these innovation outputs generate benefits that contribute to sustainable development.

Given their central role within the framework, it is helpful to point out at the outset that *innovation outputs* are seen as including new forms of governance, organization, or process work; product and service innovations; and policy innovations (Sørensen & Torfing, 2011). In the case of sustainability-oriented bottom-up initiatives—such as low impact housing developments or community composting schemes—these innovations are further characterized by the common goal of promoting sustainable development, and by a strong involvement of NPOs in the innovation process. The results are “novel bottom–up solutions for sustainable development; solutions that respond to the local situation and the interests and values of the communities involved” (Seyfang & Smith, 2007, p. 585). These are innovations that improve performance based on ecological, economic, and social criteria for the definition of such performance (Boons et al., 2013; Carrillo-Hermosilla et al., 2010) and whose characteristics may differ according to specific spatial, temporal, and cultural conditions (Boons et al., 2013).

Sustainability-oriented innovations produce both intrinsic and diffusion benefits that affect a wide range of stakeholders (Seyfang & Smith, 2007), and contribute to sustainable development through the adoption of a triple bottom line approach. Intrinsic benefits within a local community may appear, for instance, in the form of reductions in car use, increase of recycling practices, or planting trees. Within that same community, other second level intrinsic benefits follow those environmental benefits as they relate to job creation, training and skills development, and personal growth. At a third level, the positive spillovers within the community can translate into an overall improvement of a sense of community, social capital and civic engagement, and better access to services and facilities (Devine-Wright, 2006, Seyfang & Smith, 2007). Diffusion benefits, on the other hand, refer to transferring the value created by the innovators to the wider community through the mobilization of NPOs, so as to create a new system with values that differ from the mainstream, thereby generating transformations in production-consumption systems in ways that single individuals cannot sustain (Maniates, 2002). In this vein, the involvement of the NPOs in co-production processes can contribute to add value in the public sphere by increasing the direct benefit for the citizens who receive a specific public service (user value), the positive outcome for the user’s family and friends (value for wider group), social cohesion and social interaction (social value), ecological sustainability (environmental value), and democratic participation through co-planning of services with the stakeholders (political value) (Bovaird & Loeffler, 2012).

From the organizational-level perspective of the societal orientation literature, the ‘beneficiary or recipient orientation’ that is one of its components generates advantages for the beneficiaries (or customers) of the activities and/or services that are provided through the collaboration, as their needs and the related response are to be regularly monitored and adjusted accordingly. This will likely increase beneficiary response, which can be measured in different ways, including in terms of satisfaction, attendance, participation, or improvement reported by a user’s supervisor (Duque-Zuluaga & Schneider, 2008). More generally, as highlighted by Liao et al. (2001), benefits will accrue to the collaborating partners’ stakeholders whenever the former adopt the ‘stakeholder orientation’—that is, a focus on their needs—that is a key part of societal orientation.

Innovation outputs result from collaborative processes that involve partners with different identities, roles, and resources (*collaborative innovation processes*) (Sørensen & Torfing, 2011). Because of mutual learning, their interaction generates new ideas, with the overall process being facilitated by the development of forms of joint ownerships that contribute to overcome resistance, and to ensure coordination and flexible adjustment. Differences in goals and practices, often linked to different institutional logics (e.g. between businesses and NPOs), may be overcome when partners adopt a partnership logic based on “goal realignment, power rebalance and creative use of limited resources” (Yin & Jamali, 2020, p. 18). A ‘collaborative orientation’ allows NPOs to—among other benefits—increase their problem solving capabilities and improve their service delivery efficiency (Duque-Zuluaga & Schneider, 2008), which feeds into their capabilities to produce product/service as well as process innovations.

The literature on sustainability transitions (i.e. Geels, 2005; Loorbach, 2007; Rip & Kemp, 1998; Rotmans et al, 2001; Smith et al, 2005) further suggests that sustainability-oriented innovation processes are often performed within protected spaces, where the communities that promote and host such initiatives are labeled ‘strategic niches’ (Kemp et al., 1998; Seyfang & Haxeltine, 2012). The activities that characterize such niches include: the management of actors’ expectations; the promotion of networks of actors through the alignment of visions and interests towards a collective goal; and the stimulation of learning about the problems, needs and possibilities of a given solution (Kemp et al., 1998). More specifically, managing expectations relates to how niches are displayed to external audiences, and the extent to which they can deliver in terms of performance. To promote niche emergence, “these expectations should be widely shared, specific, realistic and achievable” (Seyfang & Haxeltine, 2012, p. 384). Networking activities, on the other hand, best support niches when they involve different stakeholders with their diverse stock of resources. Lastly, learning processes are considered most effective when they contribute to everyday knowledge and expertise but also, at a deeper level, to people’s questioning the assumptions and constraints of regime systems (Kemp et al., 1998).

At the same time, these collaborative innovation processes are not immune from external influences. The extent to which different actors work together and use collaboration as a vehicle for innovation may depend on a number of context-bound factors that facilitate or hamper the overall process (*drivers and barriers*). These drivers and barriers may emanate from cultural norms and values, institutional logics, inter-organizational relationships, and organizational routines (Sørensen & Torfing, 2011). Contexts with particularly high institutional complexity may push partners to adopt a ‘substitution’ rather than a ‘partnership’ logic, with the ensuing lower opportunities for value creation from the collaboration (Yin & Jamali, 2020). Unmet social needs, in particular, are not the sole grassroot-level driver: NPOs’ commitment to alternative sustainable ways of doing things is another important driver that allows the development of innovative practices based on reordered priorities and alternative values (Seyfang & Haxeltine, 2012). For instance, archetypes of socio-economic systems geared towards quality of life rather than economic growth per se (Jackson, 2004; Robertson, 1999) may find expression into initiatives like locally produced food, or the rewarding of socially reproductive labor that is not adequately valued in the traditional labor market (Seyfang, 2006). On the other hand, significant challenges may affect the diffusion of sustainability-oriented innovations (Seyfang & Haxeltine, 2012), as obstacles from the social and institutional context may hinder the adaptation of the same model to other communities, the growth of the initiative, and the substitution of unsustainable models. To support diffusion, innovators may search for public funding that is often short-term and frequently linked to constraining targets imposed by funders, rather than to the needs of the recipients. A ‘donors or resource acquisition orientation’ on the part of NPOs will translate into activities aimed at retaining or attracting financial resources (Duque-Zuluaga & Schneider, 2008), and establishing relationships with funders that are nourishing rather than constraining. A ‘competitive orientation’ (Liao et al., 2001), will allow NPOs to adequately consider the impact of potential competition among partners, for instance in service delivery or in resources’ acquisition, on the collaboration and on its (innovation-related) outcomes. More generally, a ‘stakeholder orientation’ (Liao et al., 2001) is likely to increase an NPO’s ability to consider the various and diverse needs of its stakeholders, and more appropriately manage the extent to which these needs may transform into drivers or barriers to collaborative innovation.

These collaborative innovation processes and the drivers and barriers that act upon them are embedded within institutional arenas of interaction that supply the rules, norms, routines, cognitive scripts, and discourses which define the actions of the actors, thereby creating several patterns of interaction (*institutional arenas of interaction*) (Sørensen & Torfing, 2011). These institutional arenas can be shaped by proactive forms of *metagovernance* that regulate the network of actors through both hands-off and hands-on strategies. The term ‘metagovernance’ refers, in particular, to the channels and tools used by public authorities and other actors to govern various forms of collaborative arrangements, without excessive reliance on traditional forms of command and control (Sørensen & Torfing, 2017). More specifically, it is “a specific kind of second- and third-order governance that aims to improve the functioning and capacity of relatively self-governing networks to produce governance solutions that enhance the production of public value” (Sørensen & Torfing, 2017, p. 829). The challenge for metagovernors lies in a combination of influence over the network and allowance of a certain degree of autonomy, otherwise actors may lose their enthusiasm for joint problem-solving (Cristofoli et al., 2021a). In this respect, hands-on approaches may involve the direct management of participatory engagement, whereas hands-off strategies include institutional design and network framing. Through activities aimed at stabilizing the institutional arenas, enhancing drivers, and removing barriers, the metagovernors of innovation processes within the public sphere are often public actors who have legitimacy, special resources, and capacities (Klijn & Koppenjan, 2000). Moreover, in the perspective of the collaborative innovation for sustainability literature, metagovernors are required to address challenges in the various phases of the innovation life-cycle, from start-up to diffusion and scaling (Seyfang & Smith, 2007).

## Methodology and Empirical Setting

To pursue our research aim, that is to explore the roles and mechanisms that collaborating organizations may enact to facilitate the pursuit of sustainable development, we conducted a qualitative multiple case study analysis of four sustainability-oriented innovation initiatives across Europe in the fields of energy and food consumption. This study was part of a larger EU-funded project (CASI) which focused on the assessment and management of innovation practices through a conceptual framework that was built through a shared understanding of sustainability and innovation among stakeholders (Martini et al, 2020). Within this study, we collected qualitative data from four initiatives of sustainable innovation in four different EU countries (Belgium, France, Germany and Italy).

**Fig. 2:**
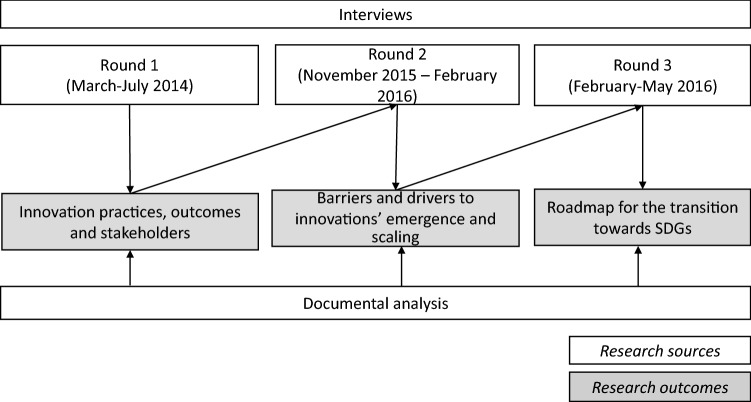
Research process

Data were collected following a three-step procedure at multiple time points between 2014 and 2016 (see Fig. 2). This longitudinal case approach is ideal to capture the richness and complexity of unfolding learning processes taking place within organizations (Yin, 2013), and to ground theory development in actual case data (Eisenhardt, 1989; Glaser & Strauss, 1967). A total of 26 semi-structured interviews were conducted involving the contact person of each innovation initiative—in all cases a person holding a management position within the key NPO in charge of promoting the innovation project—and one or two of their collaborators depending on the case. The other interviewees were representatives of the major partner organizations involved in the innovative project (between three and four depending on the project). In order to increase information reliability through triangulation (Miles & Huberman, 1994), we collected secondary data from financial reports, institutional websites, press releases, minutes of meetings, process documentation, industry reports and trade journals.

A first round of semi-structured interviews was conducted between March and July 2014, focusing on the assessment of the practices, the outcomes and the players for each case. The interviewees were asked about key objectives, origins, success factors, barriers, drivers, tensions, funding and market potential, degree of mobilization, mutual learning processes, geographical and sectoral transferability and use of assessment methods related to each sustainable innovation initiative. In addition, we investigated the overall impact of the projects on the economic, environmental and social system. A second round of interviews was conducted between November 2015 and February 2016 through half-day meetings (one for each case) with the aim to highlight the main difficulties related to the project, and identify a set of actions to overcome them. A multi-level and multi-actor perspective was adopted in defining actions, by distinguishing between management levels and stakeholders’ perspectives to which each action referred. A final round of interviews was carried out between February and May 2016 with the aim to develop an action roadmap with the innovators, consisting of activities that they would commit to accomplish to scale up the project. Interviews typically lasted approximately 2 hours, and were transcribed verbatim. Data were coded separately by the researchers based on categories that reflect the components of the framework used to guide the analysis. The results were then compared and discussed among the authors, and combined with the results of the secondary data collection, in order to build on and move beyond the informants’ descriptions, in an attempt to interpret facts and information and integrate them in an emerging and coherent framework (Lee, 1999; Miles & Huberman, 1994). Whereas the process followed a sequential path, the results from each stage were adjusted and further developed as additional sets of data made us reconsider and revise our interpretations, with the ultimate aim of improving the fit between the tentative framework and the data (Lee, 1999).

### European Cases of Sustainability-Oriented Innovation

The four cases were selected so as to ensure both the theoretical and literal replication of the results (Yin, 2013). Two out of the four initiatives (Siticibo and the Solidarity Stores) provide solutions for reducing food waste, while the other two (Fifty/Fifty and EnergyBook) aim at reducing energy consumption. Table 1 summarises the main features of the four cases as relevant to this work. In addition to the name of the project and its primary aim, the table lists the main promoter for each initiative and the multiple roles they play within the innovative process, together with the other actors involved and their respective roles; the last column highlights the sustainable development goals that are more directly linked to these initiatives.

**Table 1:** Main features of the four European initiatives of sustainable innovation

Project	Aim	Promoter	Network’s actors	Related SDG
Solidarity Stores	“fight against exclusion and sustainable reintegration without promoting assistantship”	Nationale de Développement des Epiceries Solidaires—ANDES (France): co-designer, co-implementer, accelerator	Large retailers: co-implementers National and local public administrations: financial promoters EBL agence de communication: accelerator	2. Zero Hunger 12. Responsible consumption and production
Siticibo	“daily recovery of surplus food (fresh and cooked) from the food supply chain and its redistribution to charitable organizations assisting deprived people”	Fondazione Banco Alimentare—FBA (Italy): co-designer, co-implementer, accelerator	Schools’ and companies’ canteen, hotels and catering companies: co-implementers Non-profits and individual families: users National governments and EU: accelerators	2. Zero Hunger 12. Responsible consumption and production
EnergyBook	“reduce schools’ energy consumption; invest in making school buildings capable to enhance energy savings, smart sharing of energy and sustainable energy”	Ilanga (Belgium): co-designer, accelerator	Bond Beter Leefmilieu: co-inventor and co-designer Communities of Balen and Kruibeke: co-designers, users, financial promoters, co-implementers	7. Affordable and clean energy
Fifty/Fifty	“save water and energy in schools; reduce CO2 emissions in schools; save money for schools and their stakeholders”	UfU (Germany): co-designer, co-implementer, accelerator	Federal Environmental Department: financial promoter Council facility management agencies: co-designers School districts: co-designers, co-implementers, users Students: co-implementers, accelerators Private companies (responsible for the maintenance of the schools’ heating systems): co-implementers	7. Affordable and clean energy

Based on the activities they carry out within the collaborative innovation processes, we label the relevant stakeholders as follows: *financial promoters*, supplying funds to support the initiatives; *co-designers*, engaged in the initial phases of the project; *co-implementers*, directly involved in the service provision process; and *accelerators*, carrying out direct and indirect activities that favour the diffusion of the innovation.

### Solidarity Stores—ANDES

Solidarity Stores are local convenience stores where low-income people who cannot afford to buy their food in traditional supermarkets, but who are, at the same time, reluctant to benefit from charity, can buy everyday food products at 10–20% of their commercial price. The Association Nationale de Développement des Epiceries Solidaires (ANDES—National Association for the Development of Solidarity Stores) is the network created in France in 2000 to promote the diffusion of these stores. The initiative has grown beyond the country’s borders, and expanded to include cooking lessons, parent-children activities, and employment reintegration workshops. With reference to the 2030 Sustainable Development Goals, the contribution from Solidarity Stores can be related to goals number 2, Zero Hunger (end hunger, achieve food security and improved nutrition and promote sustainable agriculture) and 12, Responsible consumption and production (ensure sustainable consumption and production patterns). ANDES’ Solidarity Stores, in fact, aim at making the consumption of fruit and vegetables accessible to people suffering from economic fragility; at the same time, they reduce food waste by promoting the consumption of non-marketable but still consumable products.

### Siticibo—Banco Alimentare

Fondazione Banco Alimentare Onlus (FBA—Food Bank Foundation) collects the production surplus of the food supply chain and, through its network of 21 local food banks, distributes it to 8.898 charitable organizations that assist the needy all over Italy. In addition to its main collection program, in 2003 FBA launched a program called ‘Siticibo’ with the aim to recover and distribute fresh and cooked food products discarded by hotels, hospitals, schools and companies, which are to be consumed within 24 h and must be transported by refrigerated vans. As in the case of the Solidarity Stores, the goals number 2, Zero Hunger and 12, Responsible consumption and production, are those that can be fulfilled through initiatives such as Siticibo. These aims are accomplished, respectively, through the daily re-distribution of cooked food to soup kitchens and nonprofit organization engaged in poverty alleviation, and through the recovery of non-marketable but consumable fresh food.

### EnergyBook—Ilanga

EnergyBook is a co-operative investment system in which parents and people from the neighbourhood invest in making school buildings more energy-efficient. Citizens, local administrations, owners of school buildings or other buildings work with coaches supplied by Ilanga and Bond Beter Leefmilieu (Union for a Better Environment), two think-tanks located in Belgium where people with expertise in energy and finance formulate solutions aimed at increasing the energy efficiency of buildings. With reference to the 2030 SDGs, the contribution from EnergyBook can be linked to goal number 7, Affordable and clean energy (ensure access to affordable, reliable, sustainable and modern energy for all).

### Fifty/Fifty—UfU

Fifty/Fifty is an initiative promoted by the German Unabhängiges Institut für Umweltfragen (UfU Independent Institute for Environmental Issues), a private research and innovation support organization, and implemented by over 3,500 schools in Germany. Participating schools receive 50% of the energy costs saved through conscious usage, to be used at their discretion; the other 50% remain with the school district. As in the case of EnergyBook, the SDG number 7, Affordable and clean energy, is directly pursued by Fifty/Fifty partners.

## European Cases of Collaborative Innovation for Sustainable Development

Based on the framework presented in section two, this section first describes the innovation outputs and their related benefits in the four cases, which are then followed by the roles and the activities of the metagovernors, and subsequently by the collaborative innovation processes and their related drivers and barriers. Table 2 summarises the main components of the framework, describes the focus of the analysis in our cases for each of these components, and outlines the main results.

**Table 2:** Operationalization of Sørensen and Torfing (2011)’s model for the analysis of collaborative innovations for sustainability

Model’s dimension	Empirical research focus	Integrations to Sørensen and Torfing (2011)’s model	Main results
Innovation outputs: Type of innovation	Identification of the primary and ancillary project innovations as they relate to product, service, process, governance model, policy	Seyfang and Smith (2007)	Additional products / services / activities are introduced together with the main innovation These different types of innovation all respond to the needs of vulnerable citizens or address environmental sustainability issues (fall into the public interest sphere)
Innovation outputs: Innovation benefits	Analysis of both intrinsic and diffusion benefits with specific focus on direct beneficiaries and wider groups (linked to direct users) and on the social, political, and environmental dimensions	Seyfang and Smith (2007) Devine-Wright (2006) Bovaird and Loeffler (2012) Duque-Zuluaga and Schneider (2008) Liao et al. (2001)	Intrinsic benefits for users and wider groups (e.g. family and friends) Extrinsic benefits: jointly reached in terms of environmental, social and political value. Environmental value as driver and consequence of the innovation
Metagovernance	Identification of hands-on and hands-off strategies through the analysis of the metagovernor’s resources and capacities, with a specific focus on the roles played by actors in the phases of both innovation start-up and diffusion	Seyfang and Smith (2007) Seyfang and Haxeltine (2012)	Hands-on strategies: NPOs provide project orchestration and contribute to project design and implementation Hands-off strategies: reduction of institutional barriers and promotion of a social and environmental sustainability culture with specific attention to the wellbeing of future generations
Institutional arenas of interaction: collaborative processes	Collaboration’s management and governance: analysis of (1) management of actors’ expectations; (2) network promotion; (3) learning stimulation	Kemp et al. (1998) Seyfang and Haxeltine (2012) Yin and Jamali (2020) Duque-Zuluaga and Schneider (2008) Liao et al. (2001)	Management of expectations: communication effort to present innovation for sustainability as a win–win solution Networking activities: involvement of different stakeholders with progressive partnership enlargement; combination of personnel from different organizations and with various backgrounds and skills Learning stimulation: first-order technological learning; additional learning processes in terms of enhanced awareness of long-term environmental effects
		Institutional arenas of interaction: drivers and barriers	Identification of potential drivers and barriers to the innovation’s implementation and diffusion, such as: presence of unmet social needs; extent of NPOs’ commitment; social and institutional conditions; and resources availability

### The Outputs of Collaborative Innovations

The outputs of the collaborative processes under study take the form of service, process, and organizational innovations, as well as innovative governance models, often with multiple types of innovation outputs within the same case.

The innovation introduced by Siticibo, for instance, is twofold. The first concerns the creation of an innovative service that was not available beforehand. The food banks belonging to the FBA network, in fact, previously provided non-profit organizations only with packaged food retrieved from large retailers or from the food industry. This newly introduced service innovation adds the distribution of discarded cooked food, collected from the canteens of schools, companies, and other organizations, to kitchens and charitable entities. Before the introduction of this service, unconsumed cooked food was bound to be thrown away because of healthcare safety reasons, with a consequent significant amount of waste. A solution, therefore, had to be found to address these safety concerns, which prompted Siticibo to engage with the challenge and concurrently introduce a process innovation: *“*with the support of Milan’s Polytechnic, we developed and adopted a new procedure to treat the food, so that we can preserve it and transport it in the appropriate way, and then it can be processed in the kitchens of the receiving charitable organizations”.

In the case of the Solidarity Stores, the innovation concerns the introduction of a new service addressing the primary needs of the less well-off directly through its stores, without the intermediation of charities, as in the case of traditional food banks. This service caters to the needs of individuals who may not resort to food banks because of the associated stigma, or who are experiencing financial difficulties without being extremely impoverished. Being able to purchase food, instead of receiving it as a donation, allows these consumers to feel they are participating in a market transaction, and therefore retain their dignity. As noted by one interviewee: “we work with community partners to minimize the barriers that may prevent individuals from seeking these kinds of services”. At the same time, the retailing activity is a means to channel more extensive solidarity actions: customers are encouraged to share their concerns and are provided with advice on how to seek help and improve their self-esteem. In order to increase these individuals’ social inclusion, and to develop their skills and competences, further collateral activities are promoted within the stores, such as cooking lessons, and parents-children activities.

EnergyBook and Fifty/Fifty display organizational and governance innovations. Both cases highlight an innovative and sustainable way to render schools self-sufficient in energy production (organizational innovation), by involving local communities simultaneously as owners and consumers (governance innovation), thereby promoting new means of citizens’ engagement. In the case of Fifty/Fifty: “schools receive 50% of the energy costs that they saved because of conscious usage of their energy and water, and they can use them at their discretion, while the other 50% remains available to the school district”. School managers together with users—pupils, teachers, and administrative staff—are directly involved in innovation implementation, which increases their commitment and sense of ownership while also educating them in the direction of a more sustainable lifestyle. This process of increased awareness and learning further extends to the pupils’ families, which facilitates its diffusion within the local community. In the case of EnergyBook there is also a dimension of service and process innovation: citizens can contact Ilanga to ask for guidance and support in their plan to make (school) buildings more energy-efficient; Ilanga coordinates the process, and helps with communication and stakeholder gathering (citizens, local administrations, owners, electricity transmission system operators etc.). Within EnergyBook, citizens are brought together to think about how they can invest in making their neighborhoods more sustainable, which not only produces innovations in local governance forms, but also increases social cohesion.

Compared to other collaborative innovations promoted by public administrations, these four initiatives do not touch public services directly; rather they affect a wider concept of public sphere. In the cases of both Siticibo and the Solidarity Stores, in particular, these initiatives contribute to poverty reduction, a major responsibility of public administrations, through food provision, which is rarely managed or financed by public institutions. At the same time, the efficient and sustainable energy consumption promoted by Fifty/Fifty and EnergyBook is not a public service per se, however it affects positively the long-term collective interest that is related to environmental protection.

### Benefits of Collaborative Sustainability-Oriented Innovations

The benefits generated by collaborative sustainability-oriented innovations are both intrinsic and extrinsic (Seyfang & Smith, 2007), and affect different dimensions of the value being created (Boivard and Loeffler, 2012). At a first level, the innovations generate benefits for the users and the wider groups who are related to them. In the case of EnergyBook and Fifty/Fifty, users include students, their families and the host communities, whereas for Siticibo the primary users are the recipients of fresh and cooked food, and for the Solidarity Stores, the shops’ clients. More specifically, the value for users of Fifty/Fifty and EnergyBook is directly related to its form in terms of environmental sustainability. As one EnergyBook interviewee explains, the benefits are also extrinsic: *“citizens, together with a coach from EnergyBook, the local administration, and the owners of the school buildings are engaged in the co-creation of a more sustainable environment”*. The beneficiary and stakeholder orientation (Duque-Zuluaga & Schneider, 2008; Liao et al., 2001) here is visible in the effort to involve all the relevant stakeholder, so that their perspective and interests are taken into consideration, thereby increasing the likelihood of the project’s success. From the perspective of Ilanga, EnergyBook’s promoter, this success contributes to the achievement of their mission and effective use of the resources they invest in the collaboration. Through this initiative, stakeholders can “reduce the schools’ energy bills, and invest in school buildings to enhance energy savings, allow smart sharing of energy, and promote sustainable energy consumption”, furthermore, with Fifty/Fifty, they can “save water and energy in the public education sector, save money for schools, and reduce CO2 emissions”.

In the case of both Siticibo and the Solidarity Stores, the positive outcome on environmental sustainability is rather an indirect implication of their business model. A Siticibo interviewee explains that the positive contribution to the environment is in a way a side effect “in addition to the direct effect as a support to charities and families”, but still it is of great importance, since “discarded food in landfills would immediately begin to produce methane gas, a greenhouse gas with over 20 times the heat-trapping capacity of carbon dioxide”.

Another component of the benefits provided by these initiatives concerns the social dimension, for instance in the form of social inclusion as explained in the case of the Solidarity Stores: “Solidarity Stores (…) are effective in preserving dignity, reducing dependence on charity and relieving beneficiaries from the feeling of being indebted. Stores are places where people are listened to and helped to rebuild their relationships with society, appreciate their own value and competences and reinforce their self-esteem. Several activities are organized there, such as cooking lessons, parent-children activities, and employment reintegration workshops”. Such social value tends to be produced through ad hoc initiatives that are promoted in addition to the main innovation. This is the case, for instance, with the Solidarity Stores’ professional integration workshops, which provide practical skills while also enhancing the attendees’ motivation to search for job opportunities*.* On the other hand, the extrinsic benefits in terms of political value occur through NPOs’ involvement in the initiatives. In the case of the innovative governance models proposed by UfU and Ilanga, value creation in the political sphere is a consequence of the communities’ direct involvement in the development of a sustainable model of energy consumption. As an EnergyBook interviewee explains: “The cultural challenge in our project does not refer exclusively to environmental sustainability, but concerns also the wider problem of citizens’ direct engagement in public issues”.

### Metagovernance: Actors and Mechanisms

The metagovernance of the initiatives under study is performed by four non-profit organizations: Fondazione Banco Alimentare (FBA) for Siticibo, the Nationale de Développement des Epiceries Solidaires (ANDES) for the Solidarity Stores, Ilanga Belgium for EnergyBook, and the Unabhängiges Institut für Umweltfragen (UfU) for Fifty/Fifty. These institutions engage in both hands-on and hands-off strategies (Sørensen & Torfing, 2017) along the multiple phases of the innovation process (Seyfang & Haxeltine, 2012; Seyfang & Smith, 2007).

Hands-on strategies are performed with different intensity across the four cases, ranging from the management of collaborative processes to active involvement in the implementation of the initiative. At the managerial level, FBA, ANDES, Ilanga, and UfU play the role of the innovation orchestrator, by integrating the contributions of various stakeholders in a coherent whole, in a way that enables collaboration toward a common goal. Moreover, both FBA and ANDES are involved in the innovation’s implementation: in the case of Siticibo and of the Solidarity Stores respectively, they play the role of both inventor and co-implementer, together with the companies that provide food and, in the case of Siticibo, non-profit organizations engaged in distribution. As noted by a Solidarity Stores’ interviewee: “ANDES supplies the know-how about how to interact with low income people and families who are affected by poverty, and also sets the conditions for accessing social stores based on socioeconomic and needs-based criteria. All the parties then participate in the financing, development and opening of the new stores, including the restructuring of buildings and the provision of shop equipment”.

These organizations also perform hands-off strategies by influencing the creation of favorable institutional arenas of interactions at two different levels. At a first level, they try to reduce potential institutional barriers through open dialog with national and European actors. As explained by FBA’s President: “The role of both EU and local governments is essential for the development of policies aimed at reducing food waste, and we perceive ourselves [FBA] as a privileged informer in the process of policy-making: the Good Samaritan Law is the best example of this process”. At a second level, they promote the development of a culture of social and environmental sustainability within the communities in which the collaborative sustainable innovations are implemented, with specific attention for the wellbeing of future generations. In the case of EnergyBook, the interviewee explained how they address the challenges of promoting a sustainability culture: “by bringing citizens together, and think about how they can invest in making their neighborhoods more sustainable, and by developing an energy cooperative in which the citizens themselves take the initiative”. In particular, the cultural transformation occurs by involving children within schools, through communication campaign programs, so as to encourage responsible energy consumption. Similar initiatives are promoted in the case of Fifty/Fifty: “we aim to promote a culture of sustainable behavior by educating students to reduce their energy and water consumption, and to act as multipliers by spreading such knowledge within their families”.

### The Institutional Arenas of Interaction: Processes and Role of the Metagovernors

The case studies highlight how the processes of network promotion, expectations’ management, and learning stimulation that characterize successful strategic niches (Kemp et al., 1998; Seyfang & Haxeltine, 2012) are primarily performed by the networks’ metagovernors.

As far as networking activities are concerned, for instance, the collaborative processes that generate sustainable innovations are prompted by the metagovernors’ attempt to involve different stakeholders at different levels, often with a progressive enlargement of the partnership, with the related increase in resources’ diversity that is considered by the literature as conducive to fruitful niche emergence. In our cases, these resources are both material (e.g. food and financial resources) and immaterial (including specialized skills and competences). In the case of the Solidarity Stores, at the outset the stores originated mainly from networks of public and non-profit organizations aimed to establish a system of free food distribution, essentially for homeless or very poor people. More recently, ANDES has worked to promote the growth of the initiative so as to include large food retailers, who played a crucial role in developing the network and allowing a rapid increase in the number and efficiency of the Solidarity Stores in France. At the micro-level, collaboration occurs among individuals, and the success of these partnerships is mainly due to an effective combination of personnel from different organizations and with various backgrounds. In the case of the Solidarity Stores, the teams consist of employees of large retailers, NPOs and public authorities who work together to launch the stores by bringing in their respective skills. Large retailers bring the specific management, logistic and sale skills that are required to meet quality standards, and provide advance financial support for warehouse and handling costs. This behavior, both at the individual and organizational level, is a reflection of the collaborative orientation (Duque-Zuluaga & Schneider, 2008) that is adopted by the metagoverning NPO as well as the other partners. The pooling of material and intangible resources allows the relevant service to be delivered in the first place, but also to increase its efficiency and quality (for instance through the involvement of large retailers in the case of the Solidarity Stores), and to build on partners’ specialized skills to foster innovation (as in the case of Siticibo’s solution to transport cooked food safely).

As for the management of expectations that is meant to be conducive to successful niche emergence, FBA begins by referring to surplus food as a ‘resource’ rather than ‘waste’. This communication effort aims at convincing food suppliers that the partnership with FBA transforms waste in resources for the recipients; at the same time, it provides a relief for the disposal of unwanted food. These savings may not be dramatic but are easily computed and therefore recognizable by partner organizations. Emphasis on surplus food as a resource instead of waste also serves the objective of aligning partners’ visions and interests that, according to the niche emergence perspective is crucial for network promotion. The network-level aim of maximizing the gains to be obtained from discarded food is aligned with the organizational-level aims of producing societal benefits while also reducing corporate costs. Similarly, in the case of EnergyBook and Fifty/Fifty, the innovation allows to reach visible and identifiable savings that are then shared by the school and the funders. Corporate costs’ reductions and energy savings are real and measurable, which makes partners’ expectations in relation to them both realistic and achievable, thereby facilitating the virtuous dynamics that characterize strategic niches. Collaboration does not necessarily concern only service provision processes: in some circumstances it involves a higher level, i.e. that of policy definition, as in the case of Siticibo. As the President of FBA explains, “the idea for Siticibo was born in 2001, inspired by a mother trying to avoid wasting excess food in schools, and was lunched in application of the Italian Law 155/2003 [the so called ‘Good Samaritan Law’]. FBA was one of the promoter of this law, which, for the first time in Europe, stated that non-profit organizations providing free distribution to food-deprived people are to be considered equal to end-consumers for the purpose of preserving, storing, and using foodstuff”. Here again the alignment of interests and objectives, together with a clear indication of the benefits that would accrue to actors at all levels, facilitated acceptance and relatively swift implementation of the innovation also at the regulatory level.

Finally, as for the kind of learning that supports the growth of niches, we see evidence of first-order learning, that is development of new knowledge, skills, and expertise (Kemp et al., 1998): this is, for instance, the case of Siticibo with the new food preservation system that allows cooked food to be transported without deteriorating. At the same time, we also see deeper learning processes with potential systemic effects (Kemp et al., 1998), as they promote greater awareness of the environmental impact of various activities. In the cases of both EnergyBook and Fifty/Fifty, for instance, participating schools commit to involve their students: Ilanga and UfU promote, in particular, the development of ad hoc training and educational modules targeted to students to increase their awareness about energy consumption and sustainability-oriented behaviors. With specific reference to Fifty/Fifty, an UfU operator suggests how “*it is innovative because it doesn’t only require changes in technology, but also in the educational curricula*”. And he adds: “*The direct involvement of students in the innovation process and the supply of lessons, project days, study groups and study trips focusing on climate and energy topics allow them to learn about energy saving practices and to transfer new knowledge to their families*”. Specific knowledge at the individual level coupled with wider receptiveness at the societal level, therefore, increase the strength of the learning processes and the likelihood that they will contribute to meaningful innovations.

### The Institutional Arenas of Interaction: Drivers and Barriers Enacted by Local Stakeholders

With reference to the drivers of these initiatives, one of EnergyBook’s interviewees identified, first of all, a growing awareness of sustainability problems among the local communities. In particular, he suggests that “in times of economic crisis, the government alone cannot provide the money and know-how that are necessary to invest in climate mitigation solutions [and] citizens are invited to proactively initiate projects”, and that with EnergyBook “*people and citizens are put at the centre*”. The presence of financial rewards for different stakeholders is another key driver. In the case of Fifty/Fifty and EnergyBook, one of the reasons for their diffusion resides in the financial interest by the community investing in the projects. The involvement of the community as financers is a key factor used by Ilanga to promote EnergyBook among schools. This resource acquisition orientation (Duque-Zuluaga & Schneider, 2008) is not directed towards donors in a strict sense, but towards citizens who may be willing to invest their excess resources in a responsible way, while also obtaining a financial return. Interviewees confirm, in fact, that “the Belgian people traditionally save a lot of money; but saving money is not economically profitable at the moment, and investing in projects that assure a return on investment is more attractive”. As a consequence, Ilanga purposefully explains to citizens that the project is cost-effective, allowing “a profit of 3 to 6% over a period of 10 to 15 years”. In the cases of both the Solidarity Stores and Siticibo, the companies that provide food surplus see their operating costs diminish due to lower management costs of inventory and waste, and often see a positive indirect impact on their revenues through an overall improvement in their reputation. According to a Solidarity Stores’ interviewee: “[our project] enhances our corporate brand image and reduces the costs of managing waste”. In the case of Siticibo, the President of FBA stressed that “the recovery of surplus food underlines the economic value of food, and donors can reduce storage and disposal fees while also putting products to good use instead of wasting them, thereby contributing to the common good of society”. The donor/resource acquisition orientation on the part of Siticibo can be seen, first, in their effort to raise companies’ awareness that their waste can be repurposed for other, socially oriented, uses; and secondly, in their linking such socially responsible behavior to cost savings for those same companies. The success of this innovation, then, is closely linked to the fact that it provides companies with the opportunity to reduce costs related to waste disposal; moreover, “the recognition of food banks’ social value has led to a strong partnership with private sector organizations, who can design their policies of corporate social responsibility as an extension of their core business”. A last important driver of collaborative innovations for sustainability may be a supportive public sector. In the case of Fifty/Fifty, Germany’s Environmental Department covers the costs that schools face during the process through the National Climate Protection Initiative for the first three years. After that period, each school district becomes responsible for keeping the project alive and ensuring its effectiveness and economic sustainability. In addition to non-economic incentives such as the positive environmental impact and the spillovers from the involvement of students, the economic incentive seems to be rather clear to public managers, who understand the opportunity to save money by embracing a strategy aimed to reduce energy and water consumption. In addition, “the fact that the initial investments for schools are covered by the national government represents another incentive for public managers as it reduces the economic risk of the initiative”.

Whereas a supportive public actor was a key driver in the case of Fifty/Fifty, Siticibo’s interviewees noted that public administrations and governmental regulations sometimes hinder the diffusion of the initiative, thereby acting as a barrier to collaborative innovation. According to a Siticibo interviewee: “sometimes the external environment does not seem to boost food surplus donation, as food donors are wary of jeopardizing their brand image. They are not willing to take on the liability risk in relation to the donated food, and there are no fiscal incentives that promote food donation”. Moreover, “regulations for the non-profit sector in Italy are often very complicated or difficult to apply”. In addition to the regulatory barriers, Siticibo’s operators identify other barriers related to the structure of the Italian food industry: “the Italian economy features a very fragmented retail industry. A few companies are leaders in every region, and then there are many small organizations operating at the local level with whom it is difficult to get in touch and begin to collaborate at the policy level. The same fragmentation is found among trade associations, which are strongly divided and are not willing to engage in mutual cooperation. The same is also true for the restaurant industry, where every organization thinks for themselves. In general, this fragmentation makes it difficult to create a network and increase the power to influence policies, at the national as well as at the European level”. These comments once again show a stakeholder orientation (Liao et al., 2001) on the part of Siticibo, highlighting the fact that such orientation can also help build a better understanding of the interests that potentially constrain the collaboration. Such improved understanding may, in turn, support the metagovernor—and/or one or more partners—in their search for an effective way to work around these constraints.

## Discussion

We have argued that NPOs may play an important role in supporting sustainable development, particularly when they are involved in various ways in the promotion of collaborative innovations. As they refer to the metagovernance of collaborative innovation processes, Sørensen and Torfing (2017, p. 830) acknowledge that “(i)t is not the prerogative of the public authorities to step into the role of metagovernor. Private actors, such as community leaders, interest organizations, and business leaders, might seek to take on this position and their success in doing so depends, among other things, on whether they possess the centrality, resources, and ability to do so”. By integrating the meso- and the micro-levels of analysis, we propose that, in the case of collaborative innovations for sustainability, such role may indeed be fruitfully played by NPOs.

The results of the analysis of the four case studies are here discussed first in relation to the contribution of collaborative innovations to SDGs (the focus of our first research question) and, second, by explaining the roles that NPOs engaged in the metagovernance of these collaborations may play in this process (which addresses our second research question). In the last part of this discussion section, we draw on our results to propose a conceptual model for the analysis of collaborative innovations for sustainability that suggests the possibility of a virtuous cycle between metagovernance, collaborative innovation processes, and value creation.

### Value Creation Through Collaborative Sustainability-Oriented Innovation

As far as the contribution of collaborative innovation to the SDGs is concerned, our analysis adds to the literature on sustainable transitions (Seyfang & Haxeltine, 2012; Smith et al., 2014) through a better understanding of how these sustainability-oriented innovations generate value, and which characteristics thereof are being created. In this vein, our results suggest that although each case focuses on one or two of the SDGs—among Zero Hunger, Responsible consumption and production, and Affordable and clean energy—their positive externalities are wider, affecting the environmental, the social and the economic dimensions of sustainable development. For instance, a positive outcome of these innovations appears to begin early in the innovative process, before the beneficial effects of services provision for users start to emerge: the NPO often acts as early-stage promoter of the innovation, and this contributes to the creation of social capital, trust, and meaningful relations long before the initiatives are implemented or the services are actually supplied. Therefore, the innovation’s benefits are multiple and possibly reinforce each other, thereby facilitating its growth across time and space. In the case of both EnergyBook and Fifty/Fifty, for instance, investing—through training—in a culture that promotes sustainability among the youth supports the creation of a sustainability culture across generations. Here, we see the immediate environmental as well as financial benefits from lower energy consumption that are enjoyed by the relevant community in the short/medium term. These immediate gains will also translate in wider and longer-term benefits, as families adopt more responsible behaviours also in other areas of their lives, and transfer this sustainability-oriented culture to their offsprings. In the case of Siticibo, the collection of discarded food that would otherwise go into incinerators has a positive impact on the environment, while also supporting the activities of NPOs that address social needs. At the same time, the collection allows companies to optimize inventory management, with positive economic implications, and to implement socially responsible practices that contribute to improved reputation and, potentially, to increasing purpose-driven behavior at the organizational level.

These initiatives, therefore, extend their scope across the environmental, social, and economic dimensions, with wider effects that reach beyond those cases where the impact tends to be limited to a single dimension. Particularly significant is the role of the financial benefits of collaborative innovation for sustainability, which may become an important driver of their diffusion and scaling-up. In the case of Fifty/Fifty, for instance, the financial benefits associated with savings from lower water and energy consumption and the related possibility to invest in other spheres are a strong incentive for scaling-up. On the other hand, even if the financial dimension may be a powerful driver, a challenge lies in showing to the relevant community the opportunity for savings and financial returns. Here the metagovernors can play a key role: rather than leveraging the intrinsic and ethical motivations of the people involved, as it happens with traditional non-profit organizations, they can build on the logic of the social enterprise, featuring a balance among the environmental, social, and economic dimensions.

Lastly, these initiatives of collaborative innovation for sustainability contribute in a fundamental way to the implementation of SDG n. 17, Partnerships for the goals, as it refers especially to the establishment of ‘multi-stakeholder partnerships that mobilize and share knowledge, expertise, technology and financial resources, to support the achievement of the sustainable development goals’ (https://sustainabledevelopment.un.org/sdg17).

### Non-profit Organizations as Metagovernors of Collaborative Innovation for Sustainability

As for the contribution of NPOs as metagovernors (Sørensen & Torfing, 2011) of multi-stakeholder and social partnerships (MacDonald et al. 2019, Yin & Jamali, 2020), our analysis adds to the interdisciplinary literature on collaboration a specific focus on the role of the actors involved. In this vein, we integrate Sørensen and Torfing (2011)’s collaborative innovation framework by focusing specifically on the NPO as a metagovernor, as opposed to the prevailing literature’s focus on public actors. Moreover, we highlight how the ‘metagovernance’ umbrella term actually includes a set of possible specific roles that the NPO may also play concurrently: the analysis leads us to identify five such roles (see Table 3).

**Table 3:** Metagovernance of collaborative innovation processes for sustainability

Role	Main activities	Examples
Cultural Spreader	Diffusion of a sustainability culture in the long term	Environmental sustainability seminars carried out at schools within Fifty/Fifty and EnergyBook respectively by UfU and Ilanga
Enabler	Creation of the conditions for collaboration through the involvement of local communities	Involvement of the community by UfU for the definition of each individual project within EnergyBook
Relational Broker	Joining resources from different actors aimed at implementing the innovative project	Network promotion by ANDES and FBA among enterprises and NPOs
Service Provider	Direct delivery of the innovative service	FBA in charge of the logistics for the transport of discarded fresh food to charities. ANDES in charge of management of the Solidarity Stores
Influencer	Support to policy makers in the development of a sustainable society	FBA as a promoter of the Good Samaritan Law

The metagovernor can play the role of a *Cultural Spreader* by promoting a culture of environmental and social sustainability among various stakeholders across time and space, thereby contributing to the generation of social and environmental value along the innovation process. For instance, UfU performs this role with the environmental sustainability seminars carried out at the schools within the Fifty/Fifty project. This actor can also play the role of the *Enabler* of the innovation co-design process, by involving the members of the local communities. These communities contribute to identify local needs and challenges, as well as the solutions that can be implemented to address them, thereby experimenting with forms of direct democracy. In the case of EnergyBook, for example, Ilanga’s coaches perform an enabling role as they build relations among citizens, the local administration, and the owners of the school buildings, so as to identify the innovative solution that is most suitable for that specific context. Similarly, the role of the *Relational Broker* also involves the promotion of networking activities among different actors, but with a specific focus on the implementation of the innovation. Metagovernors play this role when they join together resources from different actors, so as to allow the actual implementation of the innovative project. In the case of the Solidarity Stores, ANDES is engaged in the identification of new suppliers, the rearrangement of the buildings where the stores are located, and in the promotion of the initiative within the local community. At a different level, a metagovernor may become a relational broker for innovation diffusion, by promoting increasing awareness of the initiative across different communities, thereby extending value creation across space. Ilanga plays this role through the promotion of EnergyBook across Belgium. In other circumstances, the metagovernor plays the role of the *Service Provider* who’s directly involved in the provision of the innovative service. In the case of Siticibo, FBA provides the core service of fresh food transfer through its logistic infrastructure. In this role, the metagovernors create a direct benefit for users and, at the same time, they touch the environmental and economic dimensions of sustainable development by providing services (the innovation outputs) contributing to the mitigation of the environmental impact of economic activities, and, at the same time, by generating a financial pay-off for several actors. Finally, as they get involved in the development and diffusion of the innovation, the metagovernors may contribute to the formulation of public policies, thereby assuming the role of the *Influencer*. This happened for instance in the case of Siticibo, where FBA was instrumental in the promotion of the Good Samaritan Law.

### A Conceptual Model of Collaborative Innovation for Sustainability

**Fig. 3:**
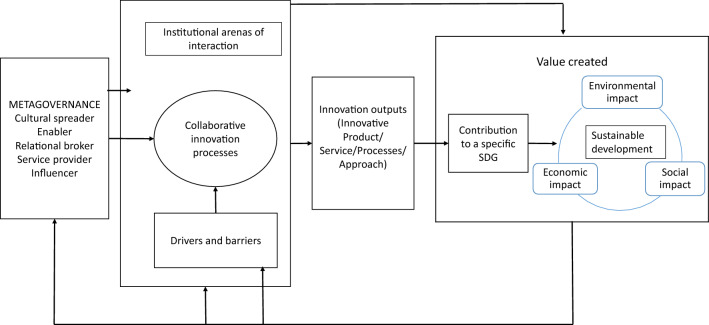
A conceptual model of collaborative innovation for sustainability

Drawing on Sørensen and Torfing (2011)’s model, integrated with contributions from with literatures on grassroots sustainable innovation and on societal orientation, and in the light of the results of our analysis, we propose a conceptual model to study collaborative innovations for sustainability that highlights the presence of a potential virtuous circle (see Fig. 3).

As in Sørensen and Torfing’s (2011) model, metagovernance affects both institutional arenas of interaction and collaborative innovation processes—with the latter being, in turn, influenced by drivers and barriers—which ultimately lead to innovation outputs. We propose an integration to this model that, first, distinguishes among five types of metagoverning roles, which in our cases appear to influence certain spheres of value creation more than others (see Table 3). Second, we propose to make explicit the value being created not only as a result of innovation outputs, but also as a result of the collaborative processes. This is most visible in the fact that the collaboration contributes to create trust and social capital early on, before the actual services (the innovation outputs) are supplied: in the EnergyBook and Fifty/Fifty cases we see an effort to create a culture of sustainability among the youth, and relatedly across generations. The benefits are multiple—as they inform the social, environmental, and financial dimensions—and reinforce each other as, for instance, increased ties and trust within the community and the visible financial rewards from energy-saving activities will likely encourage responsible behaviors also in the future. The types of value being created can also be linked directly to the dimensions of sustainable development that they contribute to support, thereby suggesting that the contribution of these initiatives to sustainable development goes well beyond the specific SDG they most directly contribute to (that is, number 2. Zero Hunger, number 12. Responsible consumption and production, and number 7. Affordable and clean energy). Our proposed framework therefore wishes to highlight that collaborative innovation processes may contribute to the various dimensions of sustainable development both directly (e.g. by fostering a culture of sustainable development among the youth) and indirectly (through the production of innovation outputs, such as a service that provides NPOs with surplus cooked food produced by companies’ canteens), and that this contribution is likely to be shaped by the role(s) taken by the metagovernor.

In addition to this set of influences, we wish to point out the feedback effects that from the value being created revert to the metagovernors and to the collaborations respectively. In the case of the metagovernors, seeing the value being created may prompt them to further promote and coordinate sustainability-oriented innovations, and/or to scale up existing ones, thereby establishing new collaborations in the process. Such visible value may take the form, for instance, of target users’ higher social inclusion through improved job market participation, or energy/water savings within the local community.

As for the effect on collaborations, the value being created may influence both their drivers and barriers. As for the drivers, different components of the value being created may contribute to increase the awareness of sustainability problems among individuals and communities: these components include, for instance, measurable improvements in pollution indicators or CO2 emissions, or reductions in food waste. More pragmatically, the financial value resulting from the collaboration may further push existing partners to collaborate, as well as attract other stakeholders. The generation of economic value may also help reduce certain barriers, as in the case of the fragmented Italian food industry in the case of Siticibo: the evidence of financial rewards may work as an incentive for companies and trade associations to set aside (part of) their conflicting interests and work together towards a common objective. Similarly, evidence of the social, environmental, and economic value being created may push public administrators to amend those regulations that act as barriers, while also establishing programs and setting aside financial resources for the promotion of sustainability-oriented partnerships.

## Conclusion

The 2030 Agenda explicitly recognizes the role to be played by cross-sector partnerships for sustainable development. However, the forms, functionings and impacts of such partnerships can be extremely varied. As noted by Yin and Jamali (2020, p. 4) in relation to social partnerships, “little has been known as to how managing the processes across the partnership dimensions may lead to value creation”.

The New Public Governance approach, and the collaborative innovation literature in particular, have shown how the wicked problems of sustainable development may be addressed through new solutions that build on partners’ diverse competences and resources (Hofstad & Torfing, 2016; Koppenjan & Klijn, 2004; Sørensen & Torfing, 2011, 2017; Van Huijstee et al., 2007), thereby shedding light on several of the aforementioned issues. On the other hand, the collaborative innovation literature mostly neglects the specific role played by non-profits within collaborations, thereby losing an opportunity to better understand possible NPO-related elements to be leveraged for greater efficacy, both from a policy perspective and from the viewpoint of the individual collaborating organizations.

The aim of our study, then, was to understand first how collaborative innovation for sustainability may actually contribute to the implementation of the Agenda 2030, and secondly which role do NPOs play within cross-sector partnerships engaged in collaborative innovation processes. To reach this objective, we conducted a multiple case study analysis of four sustainability-oriented innovation initiatives implemented across Europe in the fields of energy and food consumption.

Our results reach beyond confirming that NPOs may act as metagovernors (Sørensen & Torfing, 2017), by showing how they can actually play the central part that is often occupied by public organizations, and by suggesting a typology of the roles played by NPOs that in various ways contribute to generate innovation for sustainability. This enhanced model adds to existing frameworks by explaining how value in terms of sustainability is being created, by pointing out three major dynamics. First, collaborative innovation processes contribute to value creation both directly (e.g. by creating trust), and indirectly through the innovation outputs (e.g. a new service) that in turn generate value for users and other stakeholders. Secondly, innovation benefits for the purpose of sustainability are multiple—as they inform the social, environmental, and financial dimensions—and reinforce each other (e.g. financial and reputational rewards will encourage responsible corporate behavior). Thirdly, the value being created produces feedback effects on the metagovernors (who may be prompted to further promote sustainability-oriented innovations or scale up existing ones), and on the barriers and drivers to collaborative innovation processes (e.g. public administrators may be pushed to amend regulations that act as barriers). This model, therefore, also extends the literature on grassroots innovations for sustainability (van Lunenburg et al., 2020) as it suggests that such innovations may benefit greatly from their being promoted and implemented by partnerships between NPOs and other stakeholders. Starting from a specific aim of sustainable development—i.e. diffusion of clean energy or responsible consumption practices—grassroots innovations based on collaboration generate positive externalities at the environmental, social and economic levels, reaching far beyond their original intent. Lastly, our work adds to the societal orientation literature (Duque-Zuluaga & Schneider, 2008; Hsieh et al., 2008; Liao et al., 2001) as it highlights that all of this concept’s outward-oriented components—in addition to the NPO’s ‘collaborative orientation’—are likely to facilitate multi-stakeholder collaboration.

Joining the meso- and the micro-levels of analysis brings the benefit of integrating two different perspectives when pursuing a sustainability oriented initiative: the macro-level perspective of policy design and implementation, and the micro-level perspective of the participating organizations’ behaviour and activities. An improved understanding of the relevant roles and dynamics, therefore, allows both better institutional support and more effective collaborative behaviour, thereby improving the outlook for the implementation of Agenda 2030.

The results of this work may be of interest both from a theoretical and a practical point of view. From a theoretical perspective, this work contributes to the extant literature by developing a conceptual framework that sees collaborative innovation initiatives as drivers for the fulfilment of sustainable development, with a fundamental role to be played by NPOs not only in their design and implementation, but also in the metagovernance of the institutional arenas of collaborative interaction. From a practitioner’s viewpoint, this work provides managers of NPOs with a better understanding of the roles they may play within the innovation process. For instance, the ability to generate financial value—and to draw the attention of the relevant stakeholders to it—may strengthen the innovative process and its diffusion and scaling-up, with positive implications for value creation at different levels. In fact, part of the follow-up to our research work was the construction of a roadmap, together with the management of the organization acting as promoter/metagovernor, aimed at scaling up the innovation projects and helping them shape the mainstream approach to—in our cases—energy saving and surplus food management programs.

Our work also highlights that the four countries’ socio-economic, regulatory and economic contexts affect the innovation processes, as in the case of Siticibo facing the intricate Italian regulatory framework for NPOs, or EnergyBook benefiting from Belgians’ propensity to invest their savings. Awareness of these influences—whose examination goes beyond the scope of this paper—can help the managers of the metagovernor and of other collaborating organizations to either take advantage of or work around them.

From a policy-making perspective, the results show the opportunity for public administrations to allow NPOs to innovate trough a bottom-up approach, thereby eventually supporting sustainable development processes, and to play an active role by enabling such innovation processes while also eliminating any institutional barriers. In addition—and building on the basic idea proposed by Torfing and Triantafillou (2016) that ‘the way we shape the institutional forms of governance in the public sector affects its capacity for innovation’ (p. 3) —our results suggest that if NPOs are allowed to be metagovernors, and supported in such role, the overall system’s capacity for innovation may be enhanced. By transforming public governance in the right ways, public innovation may be boosted for the benefit of different and diverse actors.

The main limitations of this work are linked to its case study-based nature: the cases were selected through theoretical sampling, so as to explore certain elements and dynamics within a constrained comparative approach. Further research could first test the external validity of the mechanisms and dynamics highlighted by our proposed framework, by extending the analysis to other sectors, contexts, and larger samples; secondly, it could explore the extent to which such mechanisms may in fact reinforce each other and encourage a virtuous circle as our analysis suggests. Nevertheless, given the SDGs’ voluntary nature and the related weak capacity to enforce the collaborations that can foster them, our study shows that NPOs may play a crucial role not only by directly addressing local communities’ needs but also as promoters and metagovernors of collaborations among relevant stakeholders. Whereas collaboration and collaborative innovation are not without risks (Torfing & Triantafillou, 2016), they also have the potential to trigger a process of value creation that is multidimensional, and which, therefore, may contribute to sustainable development in ways that go beyond the pursuit of individual Goals. The value being created in its different forms, in turn, may act as providing incentives and reducing organizational and institutional barriers to both metagovernance and collaboration, thereby contributing to address the challenges faced by the implementation of the Agenda 2030. As of end-2021, these challenges are exacerbated by the impact of the global pandemic caused by Covid-19: at a time when publicly provided services are under considerable stress, both within and outside the healthcare sector, NPOs are seeing an increased demand for both their service delivery and innovation capabilities (Shi et al., 2020). Collaborative innovation through multi-stakeholder partnerships may be one way, among others, to answer this call.

## Footnotes

1 Figure 1 is a simplification of Sørensen and Torfing (2011)’s model, specifically as it does not include the initial conditions that influence the institutional arenas of interaction, nor the evaluation of innovation outputs.
